# Development and Validation of a Machine Learning Model to Predict Near-Term Risk of Iatrogenic Hypoglycemia in Hospitalized Patients

**DOI:** 10.1001/jamanetworkopen.2020.30913

**Published:** 2021-01-08

**Authors:** Nestoras N. Mathioudakis, Mohammed S. Abusamaan, Ahmed F. Shakarchi, Sam Sokolinsky, Shamil Fayzullin, John McGready, Mihail Zilbermint, Suchi Saria, Sherita Hill Golden

**Affiliations:** 1 Division of Endocrinology, Diabetes & Metabolism, Department of Medicine, Johns Hopkins University School of Medicine, Baltimore, Maryland; 2 Wilmer Eye Institute, Johns Hopkins University School of Medicine, Baltimore, Maryland; 3 Department of Quality Improvement and Clinical Analytics, Johns Hopkins Health System, Baltimore, Maryland; 4 Department of Biostatistics, Johns Hopkins Bloomberg School of Public Health, Baltimore, Maryland; 5 Johns Hopkins Community Physicians at Suburban Hospital, Suburban Hospital, Bethesda, Maryland; 6 Departments of Computer Science, Applied Math and Statistics, and Health Policy, Johns Hopkins University, Baltimore, Maryland

## Abstract

**Importance:**

Accurate clinical decision support tools are needed to identify patients at risk for iatrogenic hypoglycemia, a potentially serious adverse event, throughout hospitalization.

**Objective:**

To predict the risk of iatrogenic hypoglycemia within 24 hours after each blood glucose (BG) measurement during hospitalization using a machine learning model.

**Design, Setting, and Participants:**

This retrospective cohort study, conducted at 5 hospitals within the Johns Hopkins Health System, included 54 978 admissions of 35 147 inpatients who had at least 4 BG measurements and received at least 1 U of insulin during hospitalization between December 1, 2014, and July 31, 2018. Data from the largest hospital were split into a 70% training set and 30% test set. A stochastic gradient boosting machine learning model was developed using the training set and validated on internal and external validation.

**Exposures:**

A total of 43 clinical predictors of iatrogenic hypoglycemia were extracted from the electronic medical record, including demographic characteristics, diagnoses, procedures, laboratory data, medications, orders, anthropomorphometric data, and vital signs.

**Main Outcomes and Measures:**

Iatrogenic hypoglycemia was defined as a BG measurement less than or equal to 70 mg/dL occurring within the pharmacologic duration of action of administered insulin, sulfonylurea, or meglitinide.

**Results:**

This cohort study included 54 978 admissions (35 147 inpatients; median [interquartile range] age, 66.0 [56.0-75.0] years; 27 781 [50.5%] male; 30 429 [55.3%] White) from 5 hospitals. Of 1 612 425 index BG measurements, 50 354 (3.1%) were followed by iatrogenic hypoglycemia in the subsequent 24 hours. On internal validation, the model achieved a C statistic of 0.90 (95% CI, 0.89-0.90), a positive predictive value of 0.09 (95% CI, 0.08-0.09), a positive likelihood ratio of 4.67 (95% CI, 4.59-4.74), a negative predictive value of 1.00 (95% CI, 1.00-1.00), and a negative likelihood ratio of 0.22 (95% CI, 0.21-0.23). On external validation, the model achieved C statistics ranging from 0.86 to 0.88, positive predictive values ranging from 0.12 to 0.13, negative predictive values of 0.99, positive likelihood ratios ranging from 3.09 to 3.89, and negative likelihood ratios ranging from 0.23 to 0.25. Basal insulin dose, coefficient of variation of BG, and previous hypoglycemic episodes were the strongest predictors.

**Conclusions and Relevance:**

These findings suggest that iatrogenic hypoglycemia can be predicted in a short-term prediction horizon after each BG measurement during hospitalization. Further studies are needed to translate this model into a real-time informatics alert and evaluate its effectiveness in reducing the incidence of inpatient iatrogenic hypoglycemia.

## Key Points

**Question:**

Can the risk of iatrogenic hypoglycemia resulting from insulin or insulin secretagogues be predicted continuously throughout hospitalization without the use of continuous glucose monitors?

**Findings:**

In this cohort study of 54 978 admissions in a large US health care system, a stochastic gradient boosting machine learning model using 43 static and time-varying clinical predictors available in the electronic medical record accurately predicted the risk of iatrogenic hypoglycemia in a prediction horizon of 24 hours from the time of each point-of-care and serum glucose measurement throughout a patient’s hospital admission.

**Meaning:**

These findings suggest that translating this machine learning prediction model into a real-time informatics alert embedded in the electronic medical record has the potential to reduce the incidence of iatrogenic hypoglycemia, a serious adverse event.

## Teaser

This cohort study of hospitalized patients uses a machine learning model to predict the risk of iatrogenic hypoglycemia within 24 hours after blood glucose measurement during hospitalization.

## Introduction

Inpatient hypoglycemia is a prevalent and often preventable adverse event associated with increased morbidity and mortality, length of stay, readmissions, and health care expenditures.^1,2,3,4,5,6^ Considering that most patients with diabetes are hospitalized for reasons other than glucose management,^7,8^ situational unawareness of the near-term risk of iatrogenic hypoglycemia may result from competing medical priorities, lack of sufficient training in glycemic pattern recognition,^9,10,11^ or other system factors.^12^ Studies^13,14,15^ have demonstrated practitioner inertia in adjusting glucose-lowering medications before hypoglycemic events or in response to antecedent hypoglycemia.

The hospital setting poses unique challenges for the identification and prevention of hypoglycemia that results from insulin or other antihyperglycemic therapies.^7^ Many hospitalized patients have 1 or more risk factors for hypoglycemia, including advanced age, decreased renal function, liver disease, poor appetite, or nil per os (nothing by mouth) status, and some of these risk factors may change throughout a patient’s hospital stay.^1^ Changes in dextrose-containing fluids, tapering corticosteroid doses, disruption in parenteral nutrition or continuous tube feedings, mismatched timing of point-of-care blood glucose (BG) testing with insulin delivery and meal consumption, correctional scale overuse, and insulin stacking are just a few examples of the various factors that can influence the risk of iatrogenic hypoglycemia.^1^

One approach to improving situational awareness in the face of multiple dynamic data elements is to leverage the power of electronic medical record (EMR) data for real-time prediction and alerting. Previously published inpatient hypoglycemia prediction models^14,16,17,18,19^ differ with respect to cohort sample size, outcome definition, number of predictor variables, prediction horizon, statistical modeling approach, validation methods, and performance. Recently published models using logistic regression^19^ or machine learning^20^ have achieved high predictive accuracy, but the prediction horizon (during several days or entire admission) may be too long to be clinically useful in real time to guide therapeutic changes and prevent this outcome.

Because insulin dose adjustments are typically made daily for hospitalized patients based on a review of glycemic trends, an inpatient hypoglycemia prediction model that is intended to be usable in real time should predict the outcome of interest within a relatively narrow prediction horizon (ie, 24 hours) using rolling data from the EMR to account for the dynamic changes that occur throughout a patient’s hospitalization. In this study, we sought to develop a real-time prediction model for the outcome of iatrogenic hypoglycemia within a 24-hour rolling window from the time of each individual BG measurement throughout hospitalization. Moreover, given the lack of externally validated models for the outcome of iatrogenic hypoglycemia in the inpatient setting, we sought to externally validate our model by comparing its performance within different hospitals in a large health care system.

## Methods

### Study Design

The study investigators worked with Epic Clarity–certified clinical analysts to extract the data from EpicCare, the EMR system for all study hospitals. A schematic representation of the data flow from Epic Clarity (source data) to the final analytic data set is shown in eFigure 1 in the Supplement. This study followed the Transparent Reporting of a Multivariable Prediction Model for Individual Prognosis or Diagnosis (TRIPOD) reporting guideline.^21^ The study protocol was approved by the institutional review board of the Johns Hopkins School of Medicine with a waiver of informed consent because use of the large EMR data set made it impossible. Deidentified data were used during the analysis.

**Figure 1.**
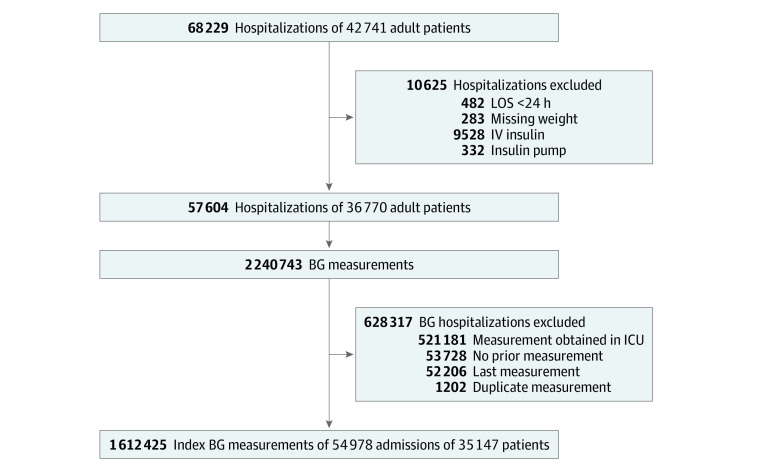
Study Flowchart All patients had at least 4 blood glucose (BG) measurements, received at least 1 U of subcutaneous insulin during admission, and were admitted to the hospital between December 1, 2014, and July 31, 2018. ICU indicates intensive care unit; IV, intravenous; and LOS, length of stay.

This retrospective cohort study consisted of EMR data obtained during routine clinical care from 5 (2 academic and 3 community) hospitals within the Johns Hopkins Health System located in Maryland and the District of Columbia. Figure 1 shows the study flowchart. The cohort consisted of hospitalized adult patients who had at least 4 BG measurements obtained during admission, received at least 1 U of subcutaneous insulin at any time during admission, and were admitted between December 1, 2014, and July 31, 2018. Admissions of patients with a length of stay of less than 24 hours, missing weight information, and treatment with intravenous insulin or insulin pumps were excluded.

The focus of this study was on noncritically ill hospitalized patients in general medical or surgical wards; therefore, BG measurements obtained at any time during admission in the intensive care unit setting were excluded. Duplicate BG measurements (ie, same value occurring at the same time) were excluded. The unit of measurement was each BG value. Because hypoglycemia as an outcome cannot be predicted for the first BG value, these values were excluded as index BG measurements. Similarly, the last BG measurement of admission was excluded because there was no future outcome to predict. After these exclusions, there were approximately 1.6 million index BG measurements of approximately 55 000 patient admissions from approximately 35 000 patients.

### Outcome

The outcome of interest was iatrogenic hypoglycemia occurring within a rolling prediction horizon of 24 hours after each index BG measurement throughout the patient’s hospital stay. Iatrogenic hypoglycemia was defined as a serum or point-of-care BG level less than or equal to 70 mg/dL (to convert to millimoles per liter, multiply by 0.0555) within the pharmacologic duration of action of administered insulins or within 24 hours of administration of a sulfonylurea or meglitinide.

### Predictors

We extracted candidate predictors from the EMR based on clinical knowledge and prior studies.^14,16,18,19,20^ All predictor variables were collected from the index admission, although some variables were from prior admissions. eTable 1 in the Supplement gives the definitions of each candidate predictor variable. Predictor variables were processed to capture accruing information in varying time frames between the time of admission and the index BG value.

### Missing Data

For most predictor variables in the data set, there were no missing observations. For missing laboratory and vital sign values, we first replaced missing observations with the median of all prior results of the variable for the patient during the admission up to the time of the index BG value. If no prior results were available, we replaced the missing result with the hospital-specific median value for all patient admissions. The counts of missing observations for each variable by hospital and the imputation methods are provided in eTable 2 in the Supplement.

### Machine Learning Models

We evaluated the performance of 4 different classification algorithms for prediction of the outcome: multivariable logistic regression, random forest classification, naive Bayes, and stochastic gradient boosting (SGB). Consistent with findings from a recent study,^20^ SGB had the highest area under the receiver operating curve on internal validation (70/30 split) using data from hospital 1; therefore, this approach was used for model development and validation.

### Model Development and Validation

Although SGB was used for development of the final prediction model, we relied on various machine learning methods for selection of our predictor variables, including univariate analysis on simple logistic regression, stepwise logistic regression and Akaike information constant, variable importance plots from random forest models, and relative influence plots in the SGB model. In selecting predictor variables, we sought to achieve the most parsimonious model possible to minimize the amount of preprocessing and computing demands that would ultimately be required if such a model were integrated into an EMR for real-time prediction.

Data from hospital 1 (largest hospital) were split into a 70% training set and 30% test set after sorting the data in chronological order by patient admission and the time of each index BG observation. The rationale for sorting data chronologically for validation was to ensure that the performance measures of the model were as conservative as possible by mimicking the real world, in which secular trends associated with unmeasured variables could influence rates of the outcome.

We developed the SGB model on the training data set. The model was constructed to predict the probability of the outcome for each index BG value. Hyperparameter tuning was performed via grid search with 10-fold cross-validation in the training set with the aim of optimizing the F1 score (harmonic mean of precision and recall). Internal validation was performed by using the training set from hospital 1 to predict the observations on the test set from hospital 1. External validation was performed by using the training set from hospital 1 to predict observations in the full data sets from hospitals 2, 3, 4, and 5.

### Statistical Analysis

All statistical analyses were performed using R statistical software, version 3.4.1 (R Foundation for Statistical Computing) and Stata software, version 15.1 (StataCorp LLC). The Caret R package was used for parameter optimizing, model training, and evaluation.^22^ Our prediction model included multiple correlated covariates at the individual level. Because our goal was to predict events at the individual level, our sampling criteria and subsequent modeling process were designed to exploit this within-patient correlation information to better predict patient-specific outcomes. Thus, our analysis did not use any clustering techniques to account for nonindependence of covariates at the patient level.

Because of the imbalance in class (positive or negative) of our outcome and the limitations of using the area under the curve alone as a comprehensive indicator of performance,^23^ we report the sensitivity (recall), positive predictive value (PPV; precision), and positive likelihood ratio (+LR; allows inferences independent of disease prevalence) together with their inverse measures (eg, specificity, negative predictive value [NPV], and negative likelihood ratio [−LR]), false-positive rate, and false-negative rate. Performance measures are reported at the probability threshold perceived by the investigators to achieve most favorable combination of sensitivity and specificity. A 2-sided *P* < .05 was considered statistically significant.

## Results

### Cohort Characteristics

This study included 54 978 admissions (35 147 inpatients; median [interquartile range] age, 66.0 [56.0-75.0] years; 27 781 [50.5%] male; 30 429 [55.3%] White) from 5 hospitals, of which 33 301 (61.6%) were academic hospital admissions and 21 677 (39.4%) were community hospital admissions. Table 1 gives the characteristics at the admission level. Despite the inclusion criterion of at least 1 U of insulin administration during hospitalization, 31 203 patients (56.8%) had no coded diagnosis of diabetes present on admission; type 2 diabetes was present in 21 660 admissions (39.4%) and type 1 diabetes in 1321 admissions (2.4%). Insulin was used at home in 13 764 admissions (25.0%) and insulin secretagogues in 7650 admissions (13.9%). There were notable differences with respect to age, race/ethnicity, weight, body mass index, length of stay, diabetes type, use of home insulin, and use of insulin secretagogues by hospital.

**Table 1.** Characteristics of the Overall Study Population and by Hospital at the Admission Level^a^

Characteristic	Entire cohort (N = 54 978)	Hospital 1 (n = 21 517)	Hospital 2 (n = 11 784)	Hospital 3 (n = 11 087)	Hospital 4 (n 2272)	Hospital 5 (n = 8318)	*P* value
Hospital type	NA	Academic	Academic	Community	Community	Community	NA
Age, median (IQR), y	66.0 (56.0-75.0)	62.0 (52.0-71.0)	64.0 (55.0-74.0)	70.0 (59.0-79.0)	70.0 (61.0-80.0)	72.0 (63.0-82.0)	<.001
Male	27 781 (50.5)	10 990 (51.1)	5770 (49.0)	5550 (50.1)	1199 (52.8)	4272 (51.4)	<.001
Race/ethnicity							
White	30 429 (55.3)	11 097 (51.6)	7454 (63.3)	6007 (54.2)	1201 (52.9)	4670 (56.1)	<.001
Black	17 806 (32.4)	8170 (38.0)	3548 (30.1)	3349 (30.2)	816 (35.9)	1923 (23.1)	
Asian	2595 (4.7)	755 (3.5)	148 (1.3)	1060 (9.6)	67 (2.9)	565 (6.8)	
Other^b^	4148 (7.5)	1495 (6.9)	634 (5.4)	671 (6.1)	188 (8.3)	1160 (13.9)	
Weight, median (IQR), kg	83.0 (68.9-100.2)	82.1 (68.0-98.9)	87.5 (72.1-105.7)	83.0 (68.0-102.1)	83.9 (70.5-99.8)	80.3 (66.7-95.7)	<.001
BMI, median (IQR)	29.0 (24.6-34.6)	28.5 (24.2-34.0)	30.5 (25.7-36.6)	29.1 (24.6-35.1)	28.8 (24.7-34.3)	28.1 (24.2-33.0)	<.001
Length of stay, median (IQR), d	5.0 (3.1-8.6)	6.0 (3.4-10.2)	4.7 (2.9-8.1)	4.7 (3.0-7.4)	4.8 (3.0-8.2)	4.3 (2.9-7.1)	<.001
Diagnosis of diabetes at admission							
None	31 203 (56.8)	13 069 (60.7)	5853 (49.7)	6400 (57.7)	1301 (57.3)	4580 (55.1)	<.001
Type 1	1321 (2.4)	423 (2.0)	294 (2.5)	378 (3.4)	45 (2.0)	181 (2.2)	
Type 2	21 660 (39.4)	7610 (35.4)	5525 (46.9)	4180 (37.7)	895 (39.4)	3450 (41.5)	
Other	794 (1.4)	415 (1.9)	112 (1.0)	129 (1.2)	31 (1.4)	107 (1.3)	
Home insulin	13 764 (25.0)	3848 (17.9)	2739 (23.2)	3680 (33.2)	978 (43.0)	2519 (30.3)	<.001
Home oral insulin secretagogues	7650 (13.9)	1737 (8.1)	1405 (11.9)	2345 (21.2)	394 (17.3)	1769 (21.3)	<.001
No. of blood glucose measurements per admission, median (IQR)	25.0 (16.0-44.0)	31.0 (18.0-55.0)	24.0 (15.0-42.0)	23.0 (15.0-36.0)	22.0 (14.0-38.0)	21.0 (14.0-34.0)	<.001

Abbreviations: BMI, body mass index (calculated as weight in kilograms divided by height in meters squared); IQR, interquartile range; NA, not applicable.

^a^ Data are presented as number (percentage) of patients unless otherwise indicated.

^b^ Includes American Indian or Alaska Native, Native Hawaiian or other Pacific Islander, unknown, or listed as *other* in the electronic medical record.

At least 1 hypoglycemic episode occurred in 8765 admissions (15.9%). At the index BG observation level, 50 345 of 1 612 425 index BG measurements (3.1%) were followed by an iatrogenic hypoglycemic episode in the next 24 hours. eTable 3 in the Supplement gives the characteristics of the cohort at the index BG level. eFigures 2 and 3 in the Supplement show the frequency distribution of iatrogenic hypoglycemia at the admission and patient levels, respectively.

### Model Specification

**Figure 2.**
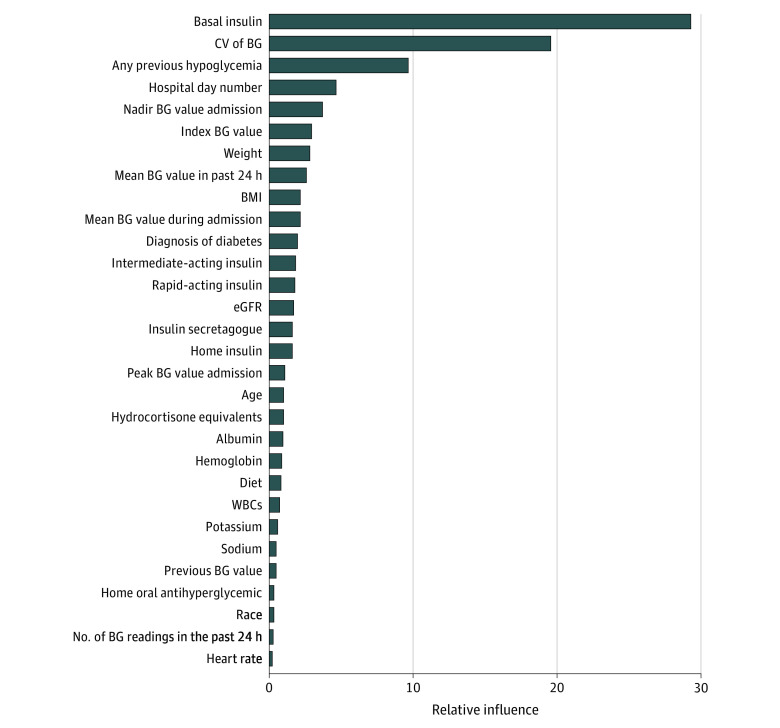
Variable Importance Plot of Top 30 Predictor Variables BG indicates blood glucose; BMI, body mass index; CV, coefficient of variation; eGFR, estimated glomerular filtration rate; WBC, white blood cell count.

**Figure 3.**
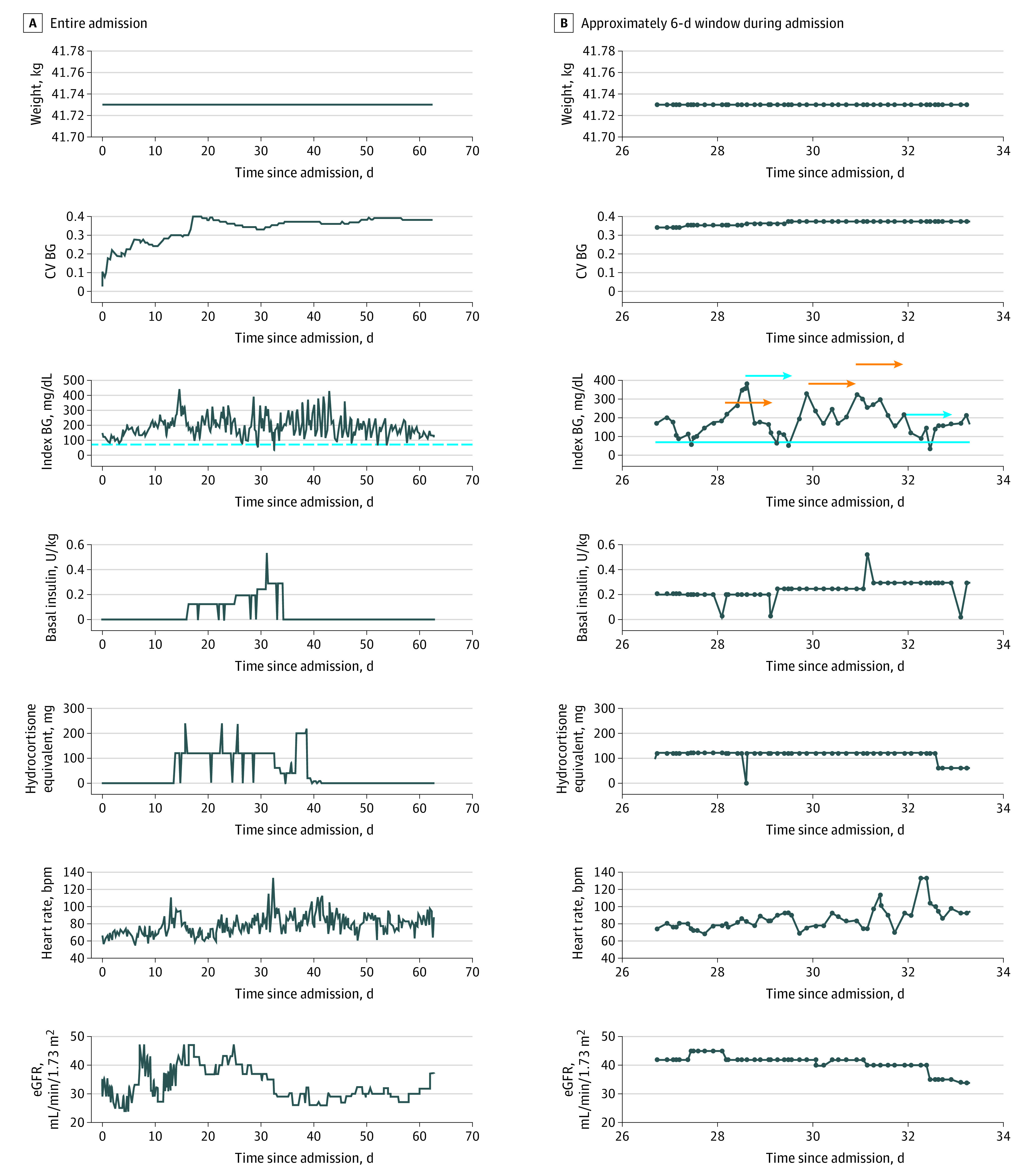
Prediction Horizon for a Sample Patient in the Data Set, Showing Only 7 of the 43 Predictor Variables in the Model The prediction model uses data of all predictor variables during hospital stay up to the time of index blood glucose (BG) value to predict outcome in the 24 hours after the index BG value. Weight is an example of a time-fixed variable, whereas all other variables shown are time varying. Orange and blue arrows indicate 24-hour prediction horizons from time of index BG values in which iatrogenic hypoglycemia is absent and present, respectively. bpm indicates beats per minute; CV, coefficient of variation; eGFR, estimated glomerular filtration rate.

The final prediction model included 43 predictor variables, of which 13 were static and 30 were time varying (eTables 1 and 4 in the Supplement). Figure 2 shows the relative importance of the top 30 predictor variables. The most important predictors were pharmacologically active basal insulin at the time of the index BG measurement, coefficient of variation of BG, any previous hypoglycemic episodes, hospital day number, nadir BG value during admission, index BG value, weight, and mean BG value in the past 24 hours. Figure 3 illustrates the dynamic nature of the prediction model using a sample patient from the cohort.

### Model Performance

Table 2 gives the model performance on internal and external validation. Our model achieved strong discrimination, with a C statistic of 0.90 (95% CI, 0.89-0.90) on internal validation and ranging from 0.86 to 0.88 on external validation; however, the high discrimination can in large part be attributed to the high prevalence of correctly predicted nonevents. On internal validation, the model achieved a PPV of 0.09 (95% CI, 0.08-0.09), NPV of 1.00 (95% CI, 1.00-1.00), +LR of 4.67 (95% CI, 4.59-4.74), −LR of 0.22 (95% CI, 0.21-0.23), false-negative rate of 18%, and false-positive rate of 18%. On external validation, the PPV ranged from 0.12 to 0.13, NPV was 0.99, +LR ranged from 3.09 to 3.89, −LR ranged from 0.23 to 0.25, false-negative rate was 18%, and false-positive rate ranged from 21% to 27%. The +LR, which is an indicator of disease likelihood that is independent of disease prevalence, was approximately 5 on internal validation, which corresponds to an approximate 30% increase in the probability of the outcome with a positive prediction.^24^ We conducted a sensitivity analysis in which we excluded observations in which any variable had a missing result and found that the model performed similarly (eTable 5 in the Supplement).

**Table 2.** Model Performance on Internal and External Validation^a^

Variable	Internal validation, hospital 1	External validation			
		Hospital 2	Hospital 3	Hospital 4	Hospital 5
C statistic	0.90 (0.89-0.90)	0.88 (0.88-0.88)	0.87 (0.86-0.87)	0.86 (0.86-0.87)	0.86 (0.86-0.86)
Probability cut point	0.444	0.516	0.513	0.478	0.442
Sensitivity	0.82 (0.81-0.83)	0.82 (0.81-0.83)	0.82 (0.81-0.83)	0.82 (0.80-0.84)	0.82 (0.81-0.83)
Specificity	0.82 (0.82-0.83)	0.79 (0.79-0.79)	0.75 (0.75-0.75)	0.75 (0.74-0.75)	0.73 (0.73-0.74)
PPV	0.09 (0.08-0.09)	0.12 (0.12-0.12)	0.13 (0.13-0.14)	0.12 (0.12-0.13)	0.12 (0.11-0.12)
NPV	1.00 (1.00-1.00)	0.99 (0.99-0.99)	0.99 (0.99-0.99)	0.99 (0.99-0.99)	0.99 (0.99-0.99)
+LR	4.67 (4.59-4.74)	3.89 (3.84-3.93)	3.28 (3.24-3.31)	3.24 (3.16-3.32)	3.09 (3.05-3.13)
−LR	0.22 (0.21-0.23)	0.23 (0.22-0.24)	0.24 (0.23-0.25)	0.24 (0.22-0.26)	0.25 (0.23-0.26)
False-negative rate, %	18	18	18	18	18
False-positive rate, %	18	21	25	25	27

Abbreviations: +LR, positive likelihood ratio; −LR, negative likelihood ratio; NPV, negative predictive value; PPV, positive predictive value.

^a^ Data are presented as number (95% CI) unless otherwise indicated.

## Discussion

In this cohort study, an SGB machine learning algorithm predicted the potentially serious outcome of iatrogenic hypoglycemia within a narrow prediction horizon of 24 hours after each BG measurement throughout hospitalization. To our knowledge, this prediction model is the first to use such a near-term prediction horizon without reliance on continuous BG monitoring, increasing the generalizability of the model for use in a large number of hospitalized patients. Unlike other machine learning models that have been developed for prediction of inpatient hypoglycemia,^14,19,20^ the narrow prediction horizon of the model in the present study allows for the possibility of short-term, continuous prediction throughout the patient’s hospital stay. Furthermore, unlike previous models that have undergone only internal validation,^20^ these findings appear to be generalizable across hospitals with heterogeneous patient populations.

Different prediction horizons can influence not only outcome prevalence (and therefore model performance) but also the ability to translate a model into a real-time alerting system. Models that use a prediction horizon of the entire admission span^20^ would be expected to have a higher outcome prevalence and potentially higher discrimination than those that use more narrow prediction horizons; although hospital-level prediction models could potentially be used to flag patients at risk for the outcome at the time of admission, they would not be useful for real-time alerting throughout a patient’s entire hospitalization. Furthermore, many inpatients experience repeated episodes of hypoglycemia during an admission; a 1-time prediction of hypoglycemia at admission would have diminished the ability to prevent repeated episodes in such patients.

Most practitioners who treat inpatients review glycemic data and adjust insulin doses at most daily; because basal insulin is usually administered once or twice daily, practitioners must be alerted to the possibility of iatrogenic hypoglycemia as soon as the risk is detected to allow them sufficient time to proactively adjust insulin doses. A prediction horizon of 24 hours after each BG measurement should generally allow sufficient lead time for practitioners to adjust antihyperglycemic therapies. Despite the low prevalence of iatrogenic hypoglycemia with this prediction horizon (approximately 3%), the prediction model nonetheless achieved +LRs of 4.67 on internal validation and 3.09 to 3.89 on external validation. As a reference, a +LR of 2 indicates small (approximately 15%), a +LR of 5 indicates moderate (approximately 30%), and a +LR of 10 indicates large (approximately 45%) increases in the probability of an outcome with a positive result.^24^ The present model had a nearly perfect NPV (given low disease prevalence), with −LRs of 0.22 to 0.25, corresponding to an approximately 30% decrease in the probability of hypoglycemia with a negative result. Because practitioners currently have no existing clinical decision support tools to assess a patient’s risk of near-term hypoglycemia on an ongoing basis in the admission, translating this prediction model into an EMR-based decision support tool could facilitate hypoglycemia risk prediction.

A challenge in developing prediction models using EMR data is the extensive amount of data processing required to create a relational database that presents observations in the same way that a practitioner would when assessing a patient’s risk of a near-term outcome through manual review of the EMR. For example, a practitioner might review insulin doses, corticosteroid doses, glycemic trends, diet orders, and laboratory data throughout the admission and from the previous 24 hours when considering the need for therapeutic changes. In the present model, extensive data processing from the EMR was required to capture pharmacologic doses of basal insulin and corticosteroids, 2 key medications that influence glucose levels. Not surprisingly, the active basal insulin dose and glycemic summary measures were the strongest predictors of near-term hypoglycemia. It was surprising to find that corticosteroid doses and kidney function were only modestly predictive. Interestingly, this machine learning approach identified some predictors (laboratory test results and vital signs) that are not traditionally known to be risk factors for hypoglycemia, and it is more likely that these predictors are associated with patient severity of illness than directly with risk of insulin or sulfonylurea-related hypoglycemia.

In recent years, EMR data mining and machine learning have been used increasingly to develop prediction models for a wide range of clinical outcomes in the inpatient setting,^25,26^ including hypoglycemia.^20^ Big data studies^18,19,27^ have achieved significantly higher discrimination (C statistics of 0.80-0.99) for prediction of iatrogenic hypoglycemia compared with previous studies^14,16,17^ that used smaller cohorts (C statistics of 0.68-0.73). In a UK sample of 32 758 admissions, Ruan et al^20^ achieved a C statistic of 0.96 using an XGBoost machine learning model with 42 predictor variables. Notably, however, their prediction horizon was the entire patient admission and achieved a sensitivity of 0.70. Furthermore, in their study, 10-fold cross-validation in a single data set was used to estimate model performance, which could overestimate the performance in a real-world setting, where secular trends cannot be accounted for. The findings of the present study suggest that the narrower prediction horizon and external validation of the model advance the work of other groups, making the model more useful at the point of care and more generalizable across hospitals. The present study used a chronological 70/30 split for validation rather than 10-fold cross-validation, which may provide more conservative estimates of model performance and better account for temporal trends than methods that randomly partition data for training and validation.

The high discrimination in this model was in large part the result of the high number of correctly predicted true negative results. Translation of this model into a real-time informatics alert would require adjustment of the probability threshold to further increase specificity to reduce alert fatigue, even at the expense of reduced sensitivity. Consider the following 2 scenarios. First, when increasing the sensitivity to 87%, specificity decreases to 77%, with a corresponding increase in the false-positive rate from 18% to 23% and a decrease in the false-negative rate from 18% to 13%. Second, conversely, reducing sensitivity to 75% results in a specificity increase to 87%, with a corresponding decrease in the false-positive rate from 18% to 13% and an increase in the false-negative rate from 18% to 25%. Consider a practitioner who is treating 10 patients with diabetes and is expected to receive 30 BG readings from these patients in a single day. An increase in the false-positive rate of 5% could mean an additional 1 or 2 unnecessary alerts per day, which could easily contribute to practitioner alert fatigue when compounded during multiple shifts, whereas an increase in the false-negative rate could mean an additional 1 or 2 missed opportunities for hypoglycemia prevention. Thus, selecting an appropriate probability threshold would need to carefully balance perceived benefit of increased sensitivity (efficacy) against reduced specificity (alert fatigue).

### Limitations

This study has limitations. It was not possible to extract information about dextrose doses because of the various numbers of medications that contain dextrose as an additive. In addition, it was not possible to capture insulin doses as additives in continuous parenteral nutrition formulations. Also, external validation was conducted at hospitals that are within the overall health system and region, and results could differ when validated in different sociodemographic populations. Finally, the model could not account for erroneous finger-stick glucose readings or distinguish between symptomatic and asymptomatic hypoglycemia. Reliance exclusively on EMR data cannot capture all the clinical information that a practitioner uses on a daily basis when making insulin dosing decisions.

## Conclusions

This study demonstrates for the first time, to our knowledge, that a machine learning algorithm has been used to predict the short-term risk of iatrogenic hypoglycemia continuously throughout hospitalization; it achieved a modest degree of accuracy without reliance on data from continuous glucose monitors. Our next step will be to embed the prediction model in our EMR system. We have recently completed a qualitative study of practitioner stakeholders to identify the optimal features and format of a clinical decision support tool based on our prediction model. Additional studies will be needed to test the real-time effectiveness of an informatics alert derived from this prediction model in reducing the incidence of this potentially serious adverse event.

## Notes

Supplement. **eTable 1.** Data Sources and Data Processing for Candidate Predictor Variables **eTable 2.** Missing Data in Training Dataset at Index BG Level **eTable 3.** Characteristics of the Overall Study Population and by Hospital at the Index BG Level **eTable 4.** Variables Used in Final Prediction Model **eTable 5.** Model Performance on Internal Validation After Dropping Observations That Included Any Variables With Missing Result **eFigure 1.** Data Flow From EMR to Final Analytic Dataset **eFigure 2.** Histogram Showing Number of Iatrogenic Hypoglycemic Episodes per Admission **eFigure 3.** Histogram Showing Number of Iatrogenic Hypoglycemic Episodes per Patient (Across Multiple Admissions)
