# Supplementary material for: Development and Validation of a Machine Learning Model to Predict Near-Term Risk of Iatrogenic Hypoglycemia in Hospitalized Patients
Source: JAMA Netw Open. 2021 Jan 8;4(1):e2030913. doi: 10.1001/jamanetworkopen.2020.30913 (PMC7794667; doi:10.1001/jamanetworkopen.2020.30913)
Supplement: Supplement. — eTable 1. Data Sources and Data Processing for Candidate Predictor Variables eTable 2. Missing Data in Training Dataset at Index BG Level eTable 3. Characteristics of the Overall Study Population and by Hospital at the Index BG Level eTable 4. Variables Used in Final Prediction Model eTable 5. Model Performance on Internal Validation After Dropping Observations That Included Any Variables With Missing Result eFigure 1. Data Flow From EMR to Final Analytic Dataset eFigure 2. Histogram Showing Number of Iatrogenic Hypoglycemic Episodes per Admission eFigure 3. Histogram Showing Number of Iatrogenic Hypoglycemic Episodes per Patient (Across Multiple Admissions) [file jamanetwopen-e2030913-s001.pdf]

Supplementary Online Content

Mathioudakis NN, Abusamaan MS, Shakarchi AF, et al. Development and validation of a machine learning model to predict near-term risk of iatrogenic hypoglycemia in hospitalized patients. *JAMA Netw Open*. 2021;4(1):e2030913. doi:10.1001/jamanetworkopen.2020.30913

eTable 1. Data Sources and Data Processing for Candidate Predictor Variables
eTable 2. Missing Data in Training Dataset at Index BG Level
eTable 3. Characteristics of the Overall Study Population and by Hospital at the Index BG Level
eTable 4. Variables Used in Final Prediction Model
eTable 5. Model Performance on Internal Validation After Dropping Observations That Included Any Variables With Missing Result
eFigure 1. Data Flow From EMR to Final Analytic Dataset
eFigure 2. Histogram Showing Number of Iatrogenic Hypoglycemic Episodes per Admission
eFigure 3. Histogram Showing Number of Iatrogenic Hypoglycemic Episodes per Patient (Across Multiple Admissions)

This supplementary material has been provided by the authors to give readers additional information about their work.

© 2021 Mathioudakis NN et al. *JAMA Network Open*.

eTable 1. Data Sources and Data Processing for Candidate Predictor Variables

Variable	Data Source	Variable Type	Definition
**Age**	Demographics	Continuous	Age on day of admission
**Sex**		Binary	0= Female, 1= Male
**Race**		Categorical	0= White, 1=Black, 2=Asian, 3= Other
**Weight**	Vitals	Continuous	Weight in kg at admission
**Body mass index (BMI), kg/m²**		Continuous	BMI in kg/m² at admission
Height		Continuous	Height in m (at admission)
**Hospital day number**	Encounter	Continuous	Day of hospitalization at time of index BG value
**Diagnosis of diabetes**	Past Medical History, Problem List, Admission Diagnosis	Categorical	
None			No ICD-9 or ICD-10 code present in any of the data sources for diabetes
Type 1 diabetes			ICD-9 codes: 250.01, 250.03 , 250.11, 250.13 , 250.21 , 250.23, 250.31, 250.33, 250.41, 250.43, 250.51, 250.53, 250.61, 250.63, 250.71, 250.73, 250.81, 250,83, 250.91, 250.93. ICD-10 codes: E10*
Type 2 diabetes			ICD-9 codes: 250.00, 250.02, 250.10, 250.12, 250.20, 250.22, 250.30, 250.32, 250.40, 250.42, 250.50, 250.52, 250.60, 250.62, 250.70, 250.72, 250.80, 250,82, 250.90, 250.92. ICD-10 codes: E11*
Other diabetes type			ICD-9 codes: 249.xx. ICD-10 codes: E08*, E09*, E13*
**Home insulin**	Medication List	Binary	Any insulin type on medication list at time of admission (1= yes, 0= no)
**Home insulin secretagogue**	Medication List	Binary	Sulfonylurea or meglitinide on medication list at time of admission (1= yes, 0= no)
**Number of home oral antihyperglycemic medications**	Medication List	Categorical	Number of home antihyperglycemic other than insulin secretagogue. GLP-1, Metformin, DPP4, SGLT2, TZD<br>None(0)= 0<br>One (1) = 1<br>Two (2) = 2 or more

© 2021 Mathioudakis NN et al. *JAMA Network Open*.

Glycemic measures			
Index BG value, mg/dl	Labs	Continuous	Value of serum or point-of-care glucose measurement (prediction horizon is within 24 hours of the time of this measurement)
**Index BG value**			Index BG value
**Coefficient of variation (CV) of BG**			Mean/SD of all BG values since admission up to and including index BG value
**Previous BG value, mg/dl**			Most recent BG measurement before the index BG value
**Average BG value since admission, mg/dl**			Average of all BG values since admission up to and including the index BG value
**Average BG value in previous 24 hrs, mg/dl**			Average of all BG values in the previous 24 hrs. up to and including the index BG value
**Nadir BG value since admission, mg/dl**			Lowest BG value since admission up to and including the index BG value
**Peak BG value since admission, mg/dl**			Highest BG value since admission up to and including the index BG value
**Number of BG readings in previous 24 hours**			Total number of BG values in 24 hours prior to index BG value (including index BG value)
BG change last to first		Continuous	Difference between last BG reading on previous calendar day and first BG reading on calendar day of index BG
BG change overnight		Continuous	Difference between morning BG reading (06:30-10:30 AM) on day of index BG and BG reading from previous night (19:01-23:59)
BG range		Continuous	Range of all BG values since admission up to index BG value
BG slope, mg/dl per hour		Continuous	(Index BG value – previous BG value) / Time interval between index BG value and previous BG value in hours
BG source		Binary	Source of index BG: 0= serum; 1 = point-of-care
**Any previous BG ≤ 70 mg/dl**		Categorical	Total number of previous BG values occurring in the inpatient setting (including prior admissions) up to and including the index BG value. Categorical Variable:<br>None (0)= 0<br>One (1)= 1<br>Two (2) =2<br>Three (3)= 3<br>Four (4)= 4<br>Five or more (5) = Greater than or equal to 5
**Time of day of index BG value**		Categorical	Categorical variable based on time of day when index BG value was resulted:

© 2021 Mathioudakis NN et al. *JAMA Network Open*.

			Overnight (0)= [24:00-06:30]<br>Morning (1) = [06:30-10:30]<br>Mid-day (2)= [10:31-15:30]<br>Evening (3)= [15:31-19:00]<br>Night (4)= [19:01-23:59]
**Insulin Secretagogue**	Medication Administration Record (MAR	Binary	Sulfonylurea or meglitinide use within 24 hrs. of index BG value (1= yes, 0= no)
**Number of inpatient antihyperglycemic medications on board**	Medication Administration Record (MAR	Categorical	Number of inpatient antihyperglycemic medications (GLP-1, Metformin, DPP4, SGLT2, TZD), other than insulin or insulin secretagogue on board at time of index BG.<br>None (0)= 0<br>One (1) = 1<br>Two (2) = 2 or more
Insulin doses on board		Continuous	
Basal insulin, units	Medication Administration Record (MAR)		Sum of all of the following:<br>• Long-acting insulins (glargine, Levemir) administered within 24 hours prior to index BG<br>• Pre-mixed insulins (70/30, 75/25) administered within 12 hours prior to index BG value<br>• Ultra-long acting insulins (degludec or U-300 glargine) administered within 36 hours prior to index BG value<br>• Concentrated insulin (U-500 regular insulin) administered within 12 hours prior to index BG value
**Basal insulin, units/kg**			Basal insulin (units) divided by admission weight (kg)
Intermediate-acting insulin, units			Sum of all the following:<br>• Neutral protamine Hagedorn (NPH) insulin doses administered within 12 hours prior to index BG<br>• Regular insulin (SQ) doses administered within 8 hours prior to index BG
**Intermediate-acting insulin, units/kg**			Intermediate-acting insulin (units) divided by admission weight (kg)
Rapid-acting insulin, units			Sum of rapid-acting insulin (aspart, lispro) administered within 5 hours prior to index BG value
**Rapid-acting insulin, units/kg**			Rapid-acting insulin (units) divided by admission weight (kg)
Systemic steroid* doses on board		Continuous	

© 2021 Mathioudakis NN et al. *JAMA Network Open*.

Hydrocortisone, mg	Medication Administration Record (MAR)		Sum of all doses of hydrocortisone administered within 12 hours prior to index BG
Cortisone, mg			Sum of all doses of cortisone administered within 24 hours prior to index BG
Prednisone, mg			Sum of all prednisone doses administered within 36 hours prior to index BG
Prednisolone, mg			Sum of all prednisolone doses administered within 36 hours prior to index BG
Methylprednisolone, mg			Sum of all methylprednisolone doses administered within 36 hours prior to index BG
Dexamethasone, mg			Sum of all dexamethasone doses administered within 72 hours prior to index BG
**Hydrocortisone equivalents, mg**			Sum of all of the following:<br>Hydrocortisone<br>Cortisone<br>Prednisone * 4<br>Prednisolone * 4<br>Methylprednisolone * 5<br>Dexamethasone * 30
**Diet**	Orders	Categorical	Active diet order at time of index BG value. Categorical variable:<br><br>Regular (0)= Regular diet.<br>Carbohydrate controlled (1)= Carb controlled, very low carb, or Ketogenic<br>Clear/full liquid (2)= clear or full liquid diet<br>Nil per os (NPO) (3)= NPO or sips and chips.<br>Tube feeding (bolus or continuous) (4)= NPO tube feeding.<br>Unknown (5) =Unknown diet
Laboratory Results**	Labs	Continuous	
**Sodium, mEq/L**			Sodium result
**Potassium, mEq/L**			Potassium result
**Estimated glomerular filtration rate (eGFR), ml/min/1.73 m^2**			Race-specific (African American vs. non-African American) eGFR result. If eGFR reported as >60 ml/min/1.73 m^2, value was replaced with age-and race-specific normal value as follows:

© 2021 Mathioudakis NN et al. *JAMA Network Open*.

			Age (years) Average eGFR<br>18-29 116<br>30-39 107<br>40-49 99<br>50-59 93<br>60-69 85<br>70+ 75
**Hemoglobin, g/dL**			Hemoglobin result
**White blood cell (WBC) count, K/cu mm**			WBC result
**Albumin, g/dL**			Albumin result
Vital signs**	Vitals	Continuous	
**Heart rate**			Heart rate
**Body temperature, F**			Temperature
**Systolic blood pressure, mmHg**			Systolic blood pressure
**Diastolic blood pressure, mmHg**			Diastolic blood pressure
**Respiratory rate**			Respiratory rate
**Surgery during admission**	OR logbook	Binary	If any surgical procedure occurred between date of admission and time of index BG value (1=yes, 0=no)
Diagnoses			
**Chronic kidney disease**	Past Medical History, Problem List, Admission Diagnosis	Binary	CKD ICD-10 code (N18*). (1= yes, 0= no)
**End stage renal disease**	Past Medical History, Problem List, Admission Diagnosis	Binary	ESRD ICD-10 codes (N18.5 or N18.6). (1= yes, 0= no)
**Dialysis**	Past Medical History, Problem List, Admission Diagnosis	Binary	Dialysis ICD-10 code (Z99.2). (1= yes, 0= no)
**Malignancy**	Admission diagnoses	Binary	Malignancy ICD-10 code (C00-C96) is a primary or secondary admission diagnosis. (1= yes, 0= no)

*Systemic refers to oral, IV, or subcutaneously administered steroids. Ophthalmic solutions and topical preparations excluded.
** Result obtained closest in time within 24 hours prior to time of index BG value, including results obtained simultaneously within index BG value

© 2021 Mathioudakis NN et al. *JAMA Network Open.*

Bold= variable included in final prediction model

© 2021 Mathioudakis NN et al. *JAMA Network Open*.

eTable 2. Missing Data in Training Dataset at Index BG Level

	Number (%) of observations missing at index BG level					How Missing Data Were Handled
Variable	Hospital 1	Hospital 2	Hospital 3	Hospital 4	Hospital 5	
BMI	383 (0.1)	562 (0.2)	346 (0.1)	82 (0.1)	329 (0.2)	Step 1: Replace with median of all prior results for patient during the admission up to time of index BG value. Step 2: If no prior results available, replace with median from entire cohort:<br>• Hospital 1= 138<br>• Hospital 2= 139<br>• Hospital 3= 139<br>• Hospital 4= 138<br>• Hospital 5= 138
Sodium, mEq/L	7132 (0.9)	5425 (1.7)	1854 (0.7)	1980 (3.5)	5137 (3.0)	Step 1: Replace with median of all prior results for patient during the admission up to time of index BG value. Step 2: If no prior results available, replace with median from entire cohort:<br>• Hospital 1= 138<br>• Hospital 2= 139<br>• Hospital 3= 139<br>• Hospital 4= 138<br>• Hospital 5= 138
Potassium, mEq/L	16008 (2.0)	10768 (3.5)	2805 (1.0)	1984 (3.6)	5583 (3.2)	Step 1: Replace with median of all prior results for patient during the admission up to time of index BG value. Step 2: If no prior results available, replace with median from entire cohort:<br>• Hospital 1= 4.2<br>• Hospital 2= 4.1<br>• Hospital 3= 4.2<br>• Hospital 4= 4.2<br>• Hospital 5= 4.1

© 2021 Mathioudakis NN et al. *JAMA Network Open*.

Albumin	99641 (12.6)	28577 (9.2)	12319 (4.4)	6683 (12.0)	24395 (14.2)	Step 1: Replace with median of all prior results for patient during the admission up to time of index BG value. Step 2: If no prior results available, replace with median from entire cohort:<br>• Hospital 1= 3.3<br>• Hospital 2= 2.9<br>• Hospital 3= 3.5<br>• Hospital 4= 3.2<br>• Hospital 5= 3.2
Hemoglobin	22006 (2.8)	7389 (2.4)	3414 (1.2)	2030 (3.6)	7171 (4.2)	Step 1: Replace with median of all prior results for patient during the admission up to time of index BG value. Step 2: If no prior results available, replace with median from entire cohort:<br>• Hospital 1= 9.8<br>• Hospital 2= 10.2<br>• Hospital 3= 10.4<br>• Hospital 4= 10.2<br>• Hospital 5= 10.4
WBC	21999 (2.8)	7361 (2.4)	3429 (1.2)	2046 (3.7)	7532 (4.4)	Step 1: Replace with median of all prior results for patient during the admission up to time of index BG value. Step 2: If no prior results available, replace with median from entire cohort:<br>• Hospital 1= 8.4<br>• Hospital 2= 8.3<br>• Hospital 3= 8.6<br>• Hospital 4= 8.7<br>• Hospital 5= 8.9
Heart rate	1019 (0.1)	657 (0.2)	250 (0.1)	51 (0.1)	222 (0.1)	Step 1: Replace with median of all prior results for patient during the admission up to time of index BG

© 2021 Mathioudakis NN et al. *JAMA Network Open*.

						value. Step 2: If no prior results available, replace with median from entire cohort:<br>• Hospital 1= 82<br>• Hospital 2= 81<br>• Hospital 3= 81<br>• Hospital 4= 80<br>• Hospital 5= 80
Temperature	1207 (0.2)	1076 (0.3)	1116 (0.4)	112 (0.2)	455 (0.3)	Step 1: Replace with median of all prior results for patient during the admission up to time of index BG value. Step 2: If no prior results available, replace with median from entire cohort:<br>• Hospital 1= 97<br>• Hospital 2= 98.1<br>• Hospital 3= 98.2<br>• Hospital 4= 98.1<br>• Hospital 5= 98.2
SBP	2399 (0.3)	647 (0.2)	267 (0.1)	49 (0.1)	219 (0.1)	Step 1: Replace with median of all prior results for patient during the admission up to time of index BG value. Step 2: If no prior results available, replace with median from entire cohort:<br>• Hospital 1= 130<br>• Hospital 2= 130<br>• Hospital 3= 131<br>• Hospital 4= 132<br>• Hospital 5= 132
DBP	2576 (0.3)	654 (0.2)	268 (0.1)	49 (0.1)	219 (0.1)	Step 1: Replace with median of all prior results for patient during the admission up to time of index BG value. Step 2: If no prior results available, replace with median from entire cohort:

© 2021 Mathioudakis NN et al. *JAMA Network Open*.

						• Hospital 1= 69<br>• Hospital 2= 70<br>• Hospital 3= 69<br>• Hospital 4= 67<br>• Hospital 5= 69
RR	1738 (0.2)	756 (0.2)	375 (0.1)	59 (0.1)	241 (0.1)	Step 1: Replace with median of all prior results for patient during the admission up to time of index BG value. Step 2: If no prior results available, replace with median from entire cohort:<br>• Hospital 1= 18<br>• Hospital 2= 18<br>• Hospital 3= 18<br>• Hospital 4= 18<br>• Hospital 5= 18
BMI= Body mass index; WBC= white blood cell count; SBP= systolic blood pressure; DBP= diastolic blood pressure; RR= respiratory rate						

© 2021 Mathioudakis NN et al. *JAMA Network Open*.

eTable 3. Characteristics of the overall study population and by hospital at the index BG level

Factor	Entire Cohort	Hospital 1 (Academic)	Hospital 2 (Academic)	Hospital 3 (Community)	Hospital 4 (Community)	Hospital 5 (Community)	p-value
Index BG observations, N	**1612425**	**791102**	**312036**	**281618**	**55860**	**171809**	
Age, years, median (IQR)	65.0 (56.0, 75.0)	63.0 (53.0, 71.0)	64.0 (55.0, 73.0)	70.0 (60.0, 80.0)	71.0 (61.0, 80.0)	72.0 (63.0, 82.0)	<0.001
Sex: Male, no.(%)	816808 (50.7%)	409726 (51.8%)	150260 (48.2%)	140930 (50.0%)	28114 (50.3%)	87778 (51.1%)	<0.001
Race, no.(%)							<0.001
White	861353 (53.4%)	394907 (49.9%)	192591 (61.7%)	148946 (52.9%)	28480 (51.0%)	96429 (56.1%)	
Black	558766 (34.7%)	309563 (39.1%)	99334 (31.8%)	88585 (31.5%)	21215 (38.0%)	40069 (23.3%)	
Asian	73214 (4.5%)	30225 (3.8%)	3303 (1.1%)	25705 (9.1%)	1656 (3.0%)	12325 (7.2%)	
Other	119092 (7.4%)	56407 (7.1%)	16808 (5.4%)	18382 (6.5%)	4509 (8.1%)	22986 (13.4%)	
Weight, Kg, median (IQR)	81.6 (67.6, 98.9)	79.8 (65.8, 96.6)	87.1 (71.7, 105.7)	81.6 (68.0, 100.2)	82.6 (68.5, 98.4)	79.4 (65.8, 95.3)	<0.001
BMI, Kg/m2, median (IQR)	28.4 (24.0, 34.2)	27.7 (23.3, 33.3)	30.4 (25.3, 36.7)	28.4 (24.3, 34.9)	28.4 (24.3, 34.1)	27.6 (23.8, 32.7)	<0.001
Length of stay, days, median (IQR)	9.6 (5.4, 18.5)	11.9 (6.6, 23.2)	8.7 (4.9, 16.9)	7.5 (4.7, 13.0)	8.7 (5.0, 16.4)	7.0 (4.2, 13.0)	<0.001
Diagnosis of diabetes at admission, no(%)							<0.001
None	849613 (52.7%)	460608 (58.2%)	135303 (43.4%)	145960 (51.8%)	30022 (53.7%)	77720 (45.2%)	
T1DM	45690 (2.8%)	17836 (2.3%)	10020 (3.2%)	11590 (4.1%)	1107 (2.0%)	5137 (3.0%)	
T2DM	690822 (42.8%)	296152 (37.4%)	163727 (52.5%)	120909 (42.9%)	23883 (42.8%)	86151 (50.1%)	
Other DM	26300 (1.6%)	16506 (2.1%)	2986 (1.0%)	3159 (1.1%)	848 (1.5%)	2801 (1.6%)	
Home insulin, no.(%)	400587 (24.8%)	150998 (19.1%)	78160 (25.0%)	93446 (33.2%)	22761 (40.7%)	55222 (32.1%)	<0.001
Home insulin secretagogue, no. (%)	194842 (12.1)	61626 (7.8)	36284 (11.6)	55443 (19.7)	9272 (16.6)	32217 (18.8)	<0.001
Number of home oral antihyperglycemic medications, no. (%)							<0.001

© 2021 Mathioudakis NN et al. *JAMA Network Open.*

None	1212825 (75.2)	645348 (81.6)	237484 (76.1)	188634 (67.0)	34661 (62.0)	106698 (62.1)	
1	327924 (20.3)	120269 (15.2)	65219 (20.9)	74773 (26.6)	16560 (29.6)	51103 (29.7)	
2+	71676 (4.4)	25485 (3.2)	9333 (3.0)	18211 (6.5)	4639 (8.3)	14008 (8.2)	
**Glycemic Measures**							
Index BG value, mg/dL, median (IQR)	155.0 (121.0, 204.0)	149.0 (118.0, 197.0)	164.0 (126.0, 216.0)	161.0 (125.0, 212.0)	158.0 (122.0, 210.0)	155.0 (122.0, 200.0)	<0.001
CV of BG, %, median (IQR)	0.3 (0.2, 0.3)	0.2 (0.2, 0.3)	0.3 (0.2, 0.3)	0.3 (0.2, 0.3)	0.3 (0.2, 0.3)	0.3 (0.2, 0.3)	<0.001
Previous BG value, mg,dL, median (IQR)	155.0 (121.0, 205.0)	149.0 (118.0, 197.0)	164.0 (127.0, 217.0)	162.0 (125.0, 213.0)	159.0 (123.0, 211.0)	156.0 (122.0, 201.0)	<0.001
Average BG value in previous 24 hrs., mg/dl, median (IQR)	158.5 (129.6, 200.8)	152.6 (125.3, 194.2)	167.8 (136.0, 212.0)	165.4 (134.7, 208.4)	163.6 (132.8, 205.4)	159.4 (132.7, 195.2)	<0.001
Average BG since admission, mg/dl, median (IQR)	162.3 (134.1, 201.8)	155.2 (129.7, 194.4)	172.5 (141.7, 214.2)	169.0 (139.2, 210.1)	168.0 (138.3, 207.0)	163.1 (138.0, 195.5)	<0.001
Nadir BG since admission, mg/dL, median (IQR)	94.0 (73.0, 121.0)	90.0 (73.0, 113.0)	98.0 (75.0, 129.0)	98.0 (73.0, 128.0)	95.0 (71.0, 124.0)	98.0 (76.0, 125.0)	<0.001
Peak BG since admission, mg/dL, median (IQR)	258.0 (199.0, 341.0)	249.0 (192.0, 337.0)	276.0 (211.0, 360.0)	264.0 (206.0, 346.0)	269.0 (208.0, 351.0)	252.0 (201.0, 319.0)	<0.001
Number of BG readings in previous 24 hours, median (IQR)	6.0 (5.0, 7.0)	6.0 (5.0, 7.0)	6.0 (5.0, 7.0)	5.0 (5.0, 6.0)	5.0 (4.0, 6.0)	5.0 (5.0, 6.0)	<0.001
Sum of all BG ≤70 mg/dL during current and prior admissions up to and including							<0.001
0	1155391 (71.7%)	580140 (73.3%)	220023 (70.5%)	186192 (66.1%)	40832 (73.1%)	128204 (74.6%)	
1	148045 (9.2%)	73267 (9.3%)	27742 (8.9%)	26529 (9.4%)	5548 (9.9%)	14959 (8.7%)	
2	80110 (5.0%)	34776 (4.4%)	16426 (5.3%)	17042 (6.1%)	3082 (5.5%)	8784 (5.1%)	
3	53728 (3.3%)	24746 (3.1%)	9263 (3.0%)	12892 (4.6%)	1679 (3.0%)	5148 (3.0%)	
4	39669 (2.5%)	16148 (2.0%)	8297 (2.7%)	9679 (3.4%)	1239 (2.2%)	4306 (2.5%)	

© 2021 Mathioudakis NN et al. *JAMA Network Open.*

5+	135482 (8.4%)	62025 (7.8%)	30285 (9.7%)	29284 (10.4%)	3480 (6.2%)	10408 (6.1%)	
Insulin secretagogue	36801 (2.3)	3499 (0.4)	5895 (1.9)	21836 (7.8)	2133 (3.8)	3438 (2.0)	
Number of inpatient antihyperglycemic medications on board, no. (%)							
None	1540555 (95.5)	776725 (98.2)	299377 (95.9)	250960 (89.1)	49912 (89.4)	163581 (95.2)	
1	66565 (4.1)	13645 (1.7)	11914 (3.8)	28026 (10.0)	5430 (9.7)	7550 (4.4)	
2+	5305 (0.3)	732 (0.1)	745 (0.2)	2632 (0.9)	518 (0.9)	678 (0.4)	
Any insulin on board at time of index BG	838027 (52.0%)	389687 (49.3%)	179244 (57.4%)	145601 (51.7%)	29825 (53.4%)	93670 (54.5%)	<0.001
**Insulin Doses on Board at time of Index BG***							
Basal insulin, unit, median (IQR)	15 (10, 27)	15 (8, 24)	20 (10, 30)	20 (10, 30)	17 (10, 30)	15 (10, 25)	<0.001
Intermediate-acting insulin, unit, median (IQR)	4 (2, 10)	12 (6, 20)	15 (10, 22)	2 (2, 6)	3 (3, 6)	10 (6, 15)	<0.001
Rapid-acting insulin doses, unit, median (IQR)	3 (2, 6)	3 (1, 6)	3 (2, 7)	3 (2, 6)	4 (2, 6)	3 (2, 6)	<0.001
Hydrocortisone equivalent doses on board at time of index BG, mg, mean (SD)	77.6 (454.5)	105.9 (562.5)	35.5 (298.1)	62.5 (338.8)	49.1 (289.5)	57.8 (312.0)	<0.001
Diet, no.							<0.001
Regular	617280 (38.3%)	332046 (42.0%)	98333 (31.5%)	104333 (37.0%)	21899 (39.2%)	60669 (35.3%)	
Carb controlled	558624 (34.6%)	198680 (25.1%)	142823 (45.8%)	119111 (42.3%)	22078 (39.5%)	75932 (44.2%)	
Clear/full liquid	104376 (6.5%)	59348 (7.5%)	10739 (3.4%)	19924 (7.1%)	4202 (7.5%)	10163 (5.9%)	
NPO	231309 (14.3%)	147033 (18.6%)	43920 (14.1%)	20549 (7.3%)	5012 (9.0%)	14795 (8.6%)	
Tube Feeding	34956 (2.2%)	25817 (3.3%)	4225 (1.4%)	3151 (1.1%)	176 (0.3%)	1587 (0.9%)	
Unknown	65880 (4.1%)	28178 (3.6%)	11996 (3.8%)	14550 (5.2%)	2493 (4.5%)	8663 (5.0%)	
Time of day of index BG value, no.							<0.001
Overnight	219536 (13.6%)	103158 (13.0%)	34931 (11.2%)	42066 (14.9%)	8484 (15.2%)	30897 (18.0%)	
Morning	383251 (23.8%)	172722 (21.8%)	90866 (29.1%)	66391 (23.6%)	13470 (24.1%)	39802 (23.2%)	
Mid-day	322394 (20.0%)	151945 (19.2%)	65213 (20.9%)	59499 (21.1%)	11388 (20.4%)	34349 (20.0%)	

© 2021 Mathioudakis NN et al. *JAMA Network Open.*

Evening	328181 (20.4%)	166603 (21.1%)	60768 (19.5%)	56514 (20.1%)	11326 (20.3%)	32970 (19.2%)	
Night	359063 (22.3%)	196674 (24.9%)	60258 (19.3%)	57148 (20.3%)	11192 (20.0%)	33791 (19.7%)	
BG ≤70 mg/dL due to insulin or SU within 24 hours after index BG, no.	50354 (3.1%)	17667 (2.2%)	10503 (3.4%)	12735 (4.5%)	2336 (4.2%)	7113 (4.1%)	<0.001
**Other laboratory results**							
Sodium, median (IQR)	139.0 (136.0, 141.0)	138.0 (136.0, 141.0)	139.0 (136.0, 141.0)	139.0 (136.0, 141.0)	138.0 (136.0, 141.0)	138.0 (135.0, 141.0)	
Potassium, median (IQR)	4.2 (3.8, 4.5)	4.2 (3.9, 4.6)	4.1 (3.8, 4.4)	4.2 (3.8, 4.5)	4.2 (3.8, 4.6)	4.1 (3.7, 4.4)	<0.001
eGFR, median (IQR)	75.0 (52.0, 93.0)	85.0 (75.0, 93.0)	75.0 (46.0, 93.0)	75.0 (42.0, 85.0)	75.0 (75.0, 85.0)	75.0 (49.0, 85.0)	<0.001
Albumin, median (IQR)	10.1 (8.6, 11.8)	3.3 (2.9, 3.8)	2.9 (2.5, 3.4)	3.5 (3.0, 3.9)	3.2 (2.7, 3.7)	3.2 (2.8, 3.7)	<0.001
Hemoglobin, median (IQR)	8.5 (6.3, 11.3)	9.8 (8.4, 11.6)	10.2 (8.7, 11.9)	10.4 (9.0, 12.0)	10.1 (8.7, 11.9)	10.4 (8.9, 12.0)	<0.001
White blood cell count, median (IQR)	3.3 (2.8, 3.7)	8.4 (6.1, 11.3)	8.3 (6.4, 10.8)	8.7 (6.5, 11.5)	8.7 (6.4, 11.8)	9.0 (6.9, 11.8)	<0.001
**Vital signs**							
Heart rate, median (IQR)	81.0 (71.0, 92.0)	82.0 (72.0, 93.0)	81.0 (71.0, 92.0)	81.0 (71.0, 92.0)	80.0 (70.0, 91.0)	80.0 (70.0, 91.0)	<0.001
Body temperature, °F, median (IQR)	98.0 (97.5, 98.4)	97.7 (97.2, 98.2)	98.1 (97.8, 98.4)	98.2 (97.8, 98.5)	98.1 (97.8, 98.5)	98.2 (97.8, 98.6)	<0.001
Systolic blood pressure, mmHg, median (IQR)	131.0 (116.0, 145.0)	130.0 (116.0, 145.0)	130.0 (116.0, 144.0)	131.0 (117.0, 145.0)	132.0 (118.0, 145.0)	132.0 (118.0, 147.0)	<0.001
Diastolic blood pressure, mmHg, median (IQR)	69.0 (61.0, 78.0)	69.0 (62.0, 78.0)	70.0 (61.0, 78.0)	69.0 (60.0, 78.0)	67.0 (59.0, 76.0)	69.0 (62.0, 77.0)	<0.001
Respiratory rate, median (IQR)	18.0 (18.0, 20.0)	18.0 (18.0, 20.0)	18.0 (18.0, 20.0)	18.0 (17.0, 20.0)	18.0 (18.0, 19.0)	18.0 (18.0, 20.0)	<0.001
Surgery during admission up to and prior to index BG, no.	692367 (42.9%)	411336 (52.0%)	109292 (35.0%)	81719 (29.0%)	21229 (38.0)	68791 (40.0%)	<0.001
Diagnoses, no. (%)							

© 2021 Mathioudakis NN et al. *JAMA Network Open.*

Chronic kidney disease	77410 (4.8)	23276 (2.9)	15459 (5.0)	19395 (6.9)	2193 (3.9)	17087 (9.9)	<0.001
End stage renal disease	30462 (1.9)	11568 (1.5)	5157 (1.7)	8442 (3.0)	967 (1.7)	4319 (2.5)	<0.001
Dialysis	35201 (2.2)	14368 (1.8)	6971 (2.2)	4500 (1.6)	940 (1.7)	8422 (4.9)	<0.001
Malignancy	104049 (6.5)	76212 (9.6)	6104 (2.0)	7894 (2.8)	2869 (5.1)	10970 (6.4)	<0.001

* results shown only for index BG value where at least insulin was on board at time of index BG value
** result obtained closest in time at or prior to index BG value
BG= blood glucose; BMI= body mass index; T1DM= type 1 diabetes mellitus; T2DM= type 2 diabetes mellitus; CV= Coefficient of variation

© 2021 Mathioudakis NN et al. *JAMA Network Open*.

eTable 4. Variables Used in Final Prediction Model

Demographics	Diagnoses/ Procedures	Glucose	Laboratory Results	Medications/Orders	Anthropo-morphometric	Vitals
Age	Diabetes type	Index BGV	Sodium	Home insulin	Weight	Heart rate
Sex	Chronic kidney disease	CV of BG	Potassium	Home insulin secretagogue	Body mass index	Body temperature
Race	ESRD	Previous BGV	eGFR	Number of home oral antihyperglycemics		SBP
Hospital day number	Dialysis	Average BGV since admission	Hemoglobin	Inpatient insulin secretagogue		DBP
	Malignancy	Average BGV in previous 24 hrs	White blood cell count	Number of inpatient antihyperglycemic medications on board at time of index BGV		RR
	Surgery during admission	Nadir BGV since admission	Albumin	Basal insulin dose on board		
		Peak BGV since admission		IA insulin dose on board		
		Number of BG readings in previous 24 hours		RA insulin dose on board		
		Number of previous BGV ≤ 70 mg/dl at any time prior to index BG		Hydrocortisone equivalent on board		
		Time of day of index BGV		Diet		

BG= blood glucose; BGV= blood glucose value; CV= coefficient of variation; eGFR= estimated glomerular filtration rate; SBP= systolic blood pressure; DBP= diastolic blood pressure; RR= respiratory rate; ESRD= end-stage renal disease; dose on board refers to active dose at time of index BGV

© 2021 Mathioudakis NN et al. *JAMA Network Open*.

eTable 5. Model performance on internal validation after dropping observations that included any variables with missing result.

	Internal Validation
C-statistic	0.90 (0.89, 0.90)
Probability cut-point	0.434
Sensitivity	0.82 (0.81, 0.83)
Specificity	0.82 (0.82, 0.82)
PPV	0.09 (0.09, 0.09)
NPV	1.00 (0.99, 1.00)
+LR	4.61 (4.54, 4.69)
-LR	0.22 (0.20, 0.23)

PPV= positive predictive value; NPV= negative predictive value; +LR= positive likelihood ratio; -LR (negative likelihood ratio). 95% confidence intervals shown in parentheses.

© 2021 Mathioudakis NN et al. *JAMA Network Open.*

eFigure 1. Data flow from EMR to final analytic dataset

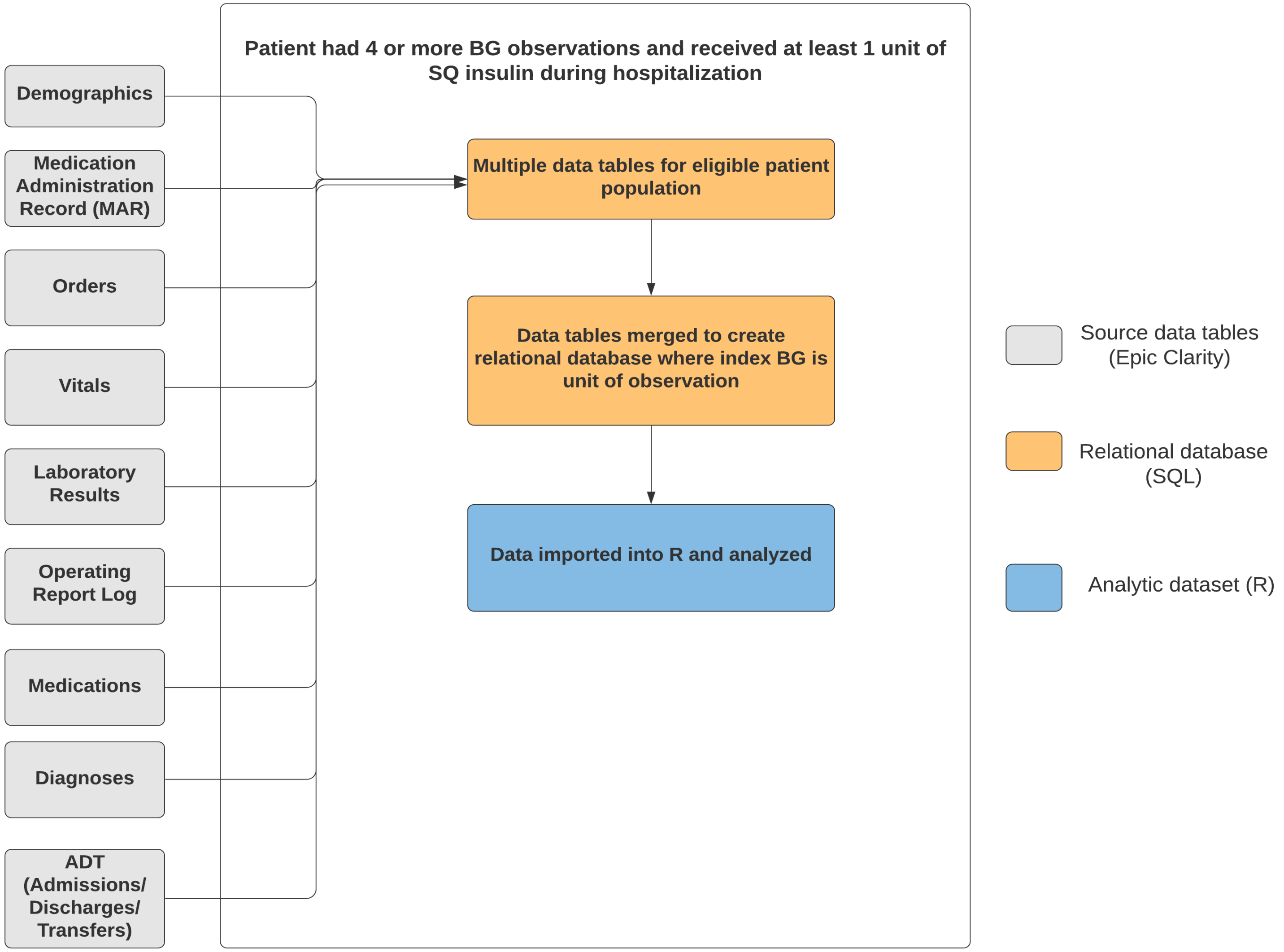

© 2021 Mathioudakis NN et al. *JAMA Network Open*.

eFigure 2. Histogram showing number of iatrogenic hypoglycemic episodes per admission.

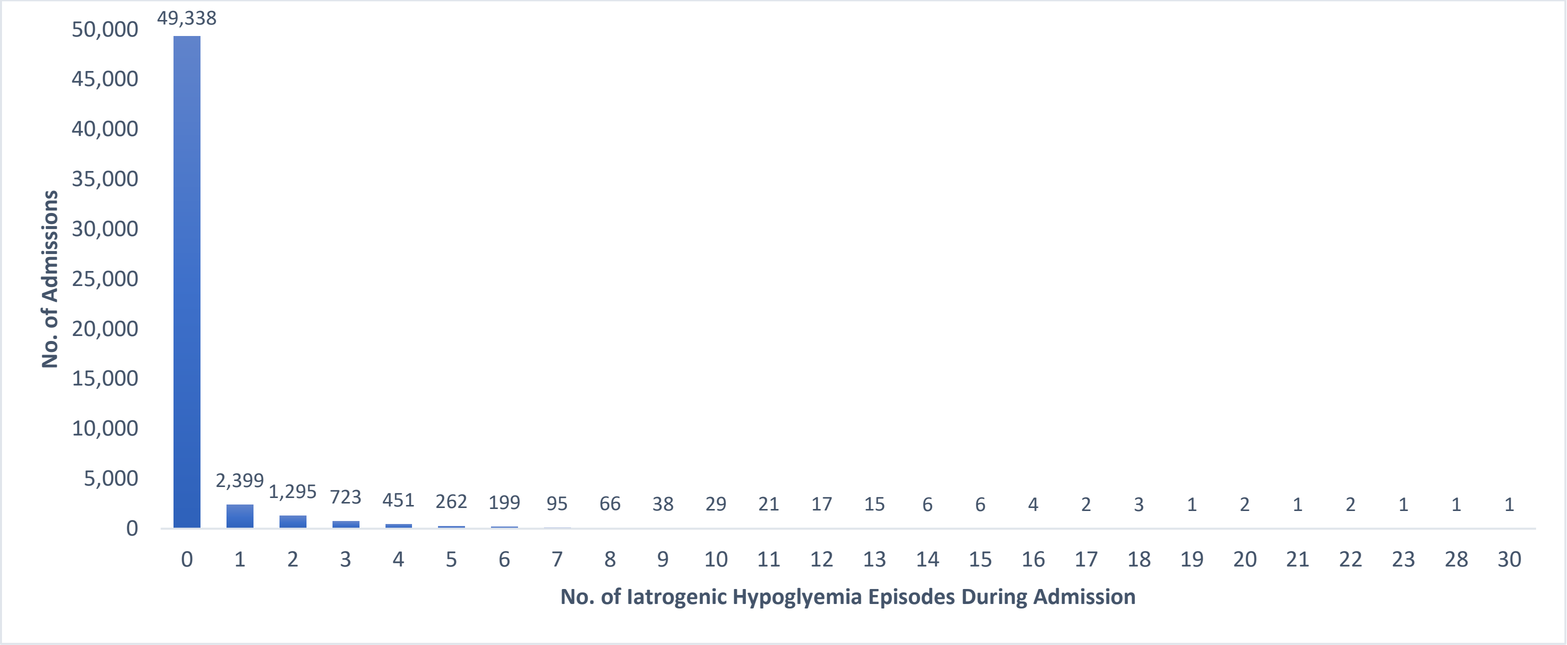

© 2021 Mathioudakis NN et al. *JAMA Network Open*.

eFigure 3. Histogram showing number of iatrogenic hypoglycemic episodes per patient (across multiple admissions).

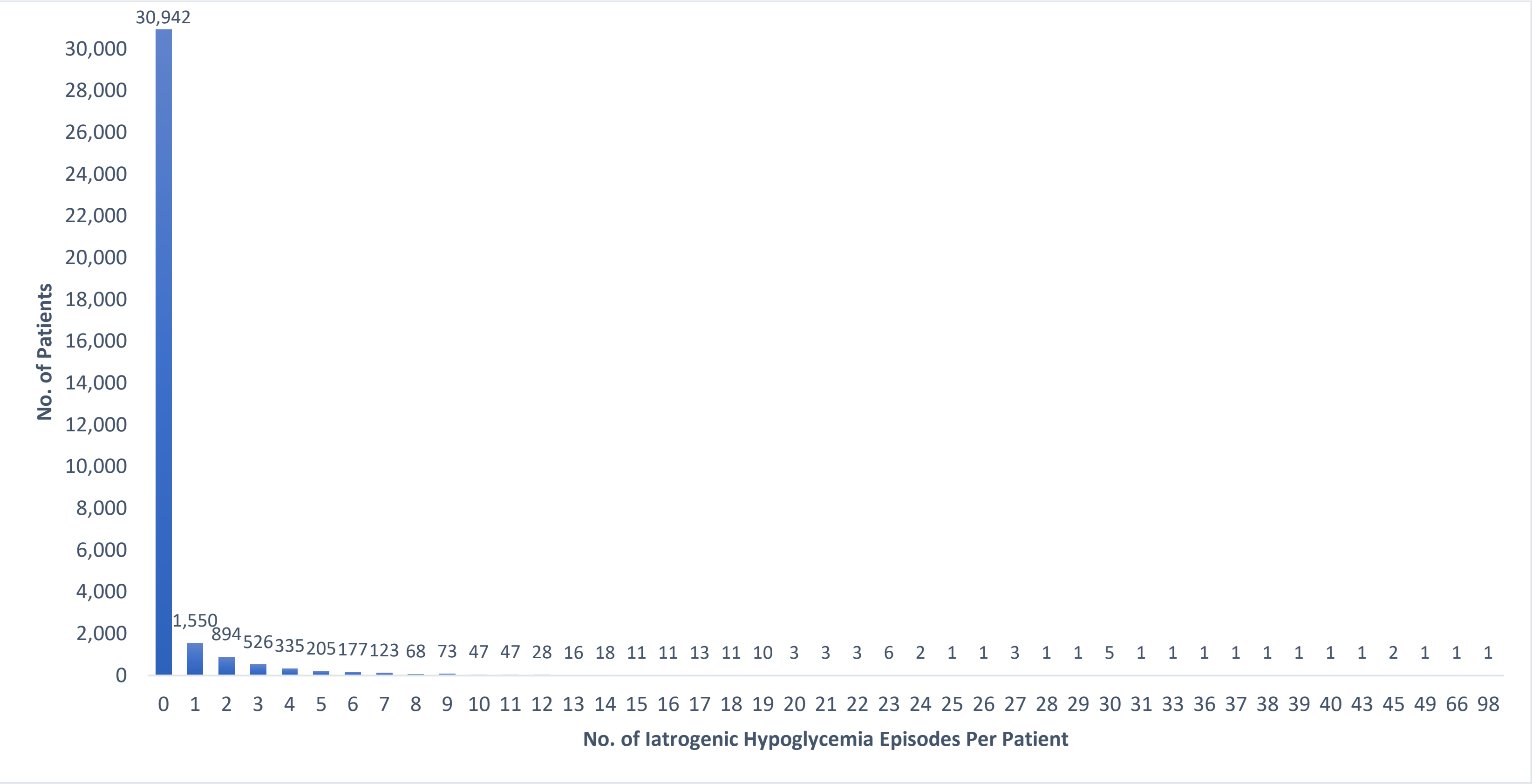

© 2021 Mathioudakis NN et al. *JAMA Network Open.*
